# Echoes of the Month

**Published:** 1924-09

**Authors:** 


### ECHOES OF THE MONTH.

### MEDICAL TREATMENT AT WEMBLEY.

It is officially stated that the number of cases medically treated at Wembley already exceeds 11,000, and though the cases have been mostly trivial, some 300 must be classed otherwise, including 12 cases of fracture, five of dislocation, and four of appendicitis. Persons old and young frequently fall into the lake, and though little harm is done they must be put to bed while their clothes are drying. Auto-intoxication due to fatigue is a common complaint among elderly folk and children, following long journeys frequently undertaken at night with little rest or sleep, combined with unsuitable and irregular meals. They are treated by rest in bed and dieting.

### HYGIENE OF PICTURE HOUSES.

Referring to the sanitation of picture houses at Blackburn, Dr. W. Allen Daley, M.O.H., records that in two cases the proprietors have put in windows so that sunlight can be admitted to the auditorium in the mornings. The resulting improvement in the hygienic conditions has been considerable. The windows also lead to a substantial monetary gain, as the cleaning can be done by natural instead of artificial light. Attention has also been paid to the angle of vision of persons occupying the front seats, to avoid the eye-strain resulting when the pictures are viewed from too short a range. The front seats have been arranged so that the horizontal angle of vision between the far edge of the picture and the observer is never less than 25 degrees, and the vertical angle between the top of the picture and the observer never less than 35 degrees.

### REFUSING TO GROW OLD.

" There is within sight," says the *Times*, " a revision of the outlook upon old age. Not much longer will this period be regarded as a gambler contemplates his unexpected winnings ; the time has arrived when the ' feeling of luck ' old folk are supposed to cherish must be exchanged for a sense of right. Old age will be counted on, like youth and middle age. It may even be pledged in advance to definite aims and purposes. This sense of a new inheritance in years, and all that it implies, has nothing in common with the old idea of growing old gracefully. Indeed it is rejection of that doctrine of passive resistance to the inevitable. To have planned the later years as the earlier are planned is to have filled them beforehand with the spirit of enterprise, which is the spirit of youth. It is to refuse to grow old at all."

### "THE HOSPITAL WHERE I WAS BORN."

Dr. Hauen Emerson, of Columbia University, New York, predicts that the familiar verse, " I remember, I remember, the house where I was born," will soon have to be altered to "The hospital where I was born," to keep pace with changing conditions. He says that in the Eastern States of America about 25 per cent. of the babies are born in hospitals, while as one goes West the number increases to 75 per cent.

### WOMEN AS GERM CARRIERS.

The Board of Control for Scotland, in the annual review of its general work, notes that there are in Scottish asylums 13 female carriers and one male carrier of enteric, and one female carrier of paratyphoid B fever. In one instance, it is stated, it seemed, failing actual proof, that infection might have been carried by a cat which was fondled by typhoid carriers and other inmates of an institution. Professor Browning, of Glasgow University, is making researches concerning carrier dangers, and, though results are not yet available, it is mentioned that much new light has been shed on the causation of these conditions which permit of individuals carrying an infectious organism in their body without exhibiting symptoms of the disease.

### A NEW ANÆSTHETIC.

It is stated that Dr. Fredet is using a new anæsthetic, " Somnifère," at the Pitié Hospital, Paris, with great success. The anæsthetic, in a dose of from ten to fifteen cubic centimetres, is injected into a vein half an hour after a preliminary hypodermic injection of morphia has been given. Long and complicated operations can be performed under this anæsthetic, it is claimed, and it is rare that it is necessary to resort to the administration of a very slight amount of chloroform. The patient remains from twenty-four to twenty-six hours in a state of coma, from which, however, it is possible to arouse him to administer nourishment. The anæsthetic is not followed by sickness, and is stated to be entirely harmless in its action.

### GOOD MILK IN GLOSSOP.

In a small urban area where the milk production is properly looked after it will probably be found that better results are secured than in a large borough ; certainly better than in rural areas where the arch-offenders are to be found. At any rate, the Sanitary Inspector for Glossop Urban District is able to report for 1923 that 51 samples of milk were bacteriologically tested and that the results were excellent. Although all the samples so tested were ordinary milk as sold to the public, in the majority of cases the quality was far above that required for " Grade A " or " Certified Milk," a state of affairs for which the farmers deserve great credit.

### WHAT IS A PANEL DOCTOR?

The De Bear Schools recently offered some free scholarships for students in Wales, Birmingham, Glasgow, Hull, and other centres, and among the questions in the general knowledge paper was one asking the candidates to write sentences containing two words, " Panel doctors." Here are some of the efforts :—" Panel doctors are different from ordinary doctors ; they are paid. Panel doctors are not fully qualified. They cannot operate on any part of the body, but one panel only. Doctors who have no motor-cars get panel pay. I understand that a panel doctor is one who attends a family for ever."

### HUMOUR IN DEATH CERTIFICATES.

A member of the staff in the American State Department of Health made a list of remarkable causes of death noticed by him during the course of his work in the Division of Vital Statistics. Among them were : " Paralises of the Hart," " Celery morbis," " Nateral causes," " Tired of living (99)," " Erley rising and marriage," " Worried to death by troublesome neighbours," " Why nobody knows," " Taking Dr. Bostwick's medicine " (this certificate was apparently signed by a competing physician).

### COST OF HEALTH SERVICES.

The Select Committee on Estimates, in their second report, urge that the Ministry of Health should consider the possibility of substituting for the percentage grants system a method which would give the Government more control over local expenditure. It will be remembered that long ago the Geddes Committee recommended the abolition of the percentage grants system, and a Committee under the presidency of Lord Meston are at present dealing with the matter. The Ministry of Health's expenditure for the current year is over £2,000,000 in excess of its expenditure for the previous year.

### ARTIFICIAL KIDNEYS.

German scientists, it is stated, are planning to use an artificial kidney invented by Dr. John J. Abel, of the Johns Hopkins Medical School, to save lives in cases of corrosive poisoning and other maladies where death is the consequence of overworking the kidneys. The false kidney is a tube of glass, which can be attached to the arm or some other part of the body. It is connected with an artery on one side and a vein on the other, so that the circulation of the blood is not impeded. The impurities are filtered by means of the artificial kidney, and are drawn from it while the real kidneys rest.

### THE MISSION TO LEPERS.

From the Quarterly Magazine of the Mission to Lepers we learn that progress is most encouraging from every point of view. A considerable amount of valuable work has been carried on in India, China, Burma, and Korea, where the Mission has homes for the care, and often the cure, of lepers. The inmates receive Christian teaching and, while no compulsion in religious matters is practised or allowed, during last year 727 were baptised in the Christian faith. The Mission's entire income, consisting of donations, legacies, government and municipal grants, totalled £78,513.

